# Reduced protein diet with near ideal amino acid profile improves energy efficiency and mitigate heat production associated with lactation in sows

**DOI:** 10.1186/s40104-019-0414-x

**Published:** 2020-02-07

**Authors:** Sai Zhang, Jay S. Johnson, Mu Qiao, Nathalie L. Trottier

**Affiliations:** 1 Department of Animal Science, Michigan State University, East Lansing, 48824 USA; 2 USDA-ARS Livestock Behavior Research Unit, West Lafayette, 47907 USA; 3 Institute of Animal Husbandry and Veterinary, Hubei Academy of Agricultural Sciences, Wuhan, 430064 China

**Keywords:** Amino acid, Energy balance, Heat production, Lactating sows, Leucine, Reduced protein diet

## Abstract

**Background:**

The study objective was to test the hypothesis that 1) lowering dietary crude protein (CP) increases dietary energetic efficiency and reduces metabolic heat associated with lactation, and 2) excessive dietary leucine (Leu) supplementation in a low CP diet decreases dietary energetic efficiency and increases metabolic heat associated with lactation.

**Methods:**

Fifty-four lactating multiparous Yorkshire sows were allotted to 1 of 3 isocaloric diets (10.80 MJ/kg net energy): 1) control (CON; 18.75% CP), 2) reduced CP with a near ideal or optimal AA profile (OPT; 13.75% CP) and 3) diet OPT with excessive Leu (OPTLEU; 14.25% CP). Sow body weight and backfat were recorded on day 1 and 21 of lactation and piglets were weighed on day 1, 4, 8, 14, 18, and 21 of lactation. Energy balance was measured on sows during early (day 4 to 8) and peak (day 14 to18) lactation, and milk was sampled on day 8 and 18.

**Results:**

Over 21-day lactation, sows fed OPT lost body weight and body lipid (*P* < 0.05). In peak lactation, sows fed OPT had higher milk energy output (*P* < 0.05) than CON. Sows fed OPTLEU tended (*P* = 0.07) to have less milk energy output than OPT and did not differ from CON. Maternal energy retention was lower (*P* < 0.05) in OPT and OPTLEU compared to CON sows, and did not differ between OPTLEU and OPT sows. Sows fed OPT had higher (*P* < 0.05) apparent energy efficiency for milk production compared to CON. Heat production associated with lactation was lower (*P* < 0.05) or tended to be lower (*P* = 0.082), respectively, in OPT and OPTLEU compared to CON sows.

**Conclusion:**

The OPT diet, in peak lactation, improved dietary energy utilization for lactation due to less urinary energy and metabolic heat loss, and triggered dietary energy deposition into milk at the expense of maternal lipid mobilization. Leucine supplementation above requirement may reduce dietary energy utilization for lactation by decreasing the energy partitioning towards milk, partially explaining the effectiveness of OPT diet over CON diets.

## Background

Lactation is an energetically costly process that depends on the sow’s ability to consume enough energy to sustain milk production. Voluntary feed intake, however, is biologically limiting [1] and the sow must rely of her body fat and protein when milk energy demand exceeds energy intake. Over the past decades, larger litter size at birth due to genetic selection have increased lactation demands [2, 3]. Strategies to improve the efficiency of dietary energy utilization are needed to sustain greater levels of milk production.

Lowering dietary crude protein (CP) in growing-finishing pigs improves energetic efficiency (i.e., retained tissue net energy:gross energy intake) due to reduced heat and urinary energy loss [4, 5]. Feeding diets with reduced CP concentrations and improved amino acid (AA) balance to lactating sows increases the efficiency of nitrogen (N) and indispensable amino acid (IDAA) utilization [6–8], and also appears to increase nutrient partitioning towards mammary metabolism [6, 8]. However, the impact of feeding such diet on energy partitioning and efficiency still need further investigation. In addition, near ideal amino acid (NIAA) profile may also reduce heat production due to changes in metabolic demand resulting from less AA destined to oxidation.

It was previously hypothesized [8] that the improved AA utilization efficiency from feeding reduced CP diets may be associated with lower intake of leucine (Leu). The premise was based on the notion that high Leu concentrations inhibit lysine (Lys) uptake in rat mammary explants [9, 10], and that potential competitive inhibition exists between Lys and Leu utilization by the mammary gland [11, 12]. In the previous study [8], addition of Leu to a reduced CP diet did not have noticeable impact on Lys efficiency, but milk yield in peak lactation was reduced and similar to that of sows fed a conventional diet, indicating some energy partitioning away from the mammary gland. In contrast, the reduced CP diet without added Leu led to greater milk yield, milk fat and lactose output and litter growth rate, but also resulted in body weight (BW) and back fat losses during peak lactation. What was noticeably greater was the milk fat concentration and milk fat yield in sows fed the reduced CP diet. Estimation of body lipid mobilization is needed to further understand the potential impact of feeding an improved AA profile on energy partitioning.

The study objective was to estimate dietary energetic efficiency, energy partitioning and heat production for lactation in sows fed a conventional diet with Leu:Lys of 1.63 (control), a reduced CP diet meeting the minimum requirement for standardized ileal digestible (SID) Leu [13] and with Leu:Lys of 1.14 (optimal), and a reduced CP diet with a SID Leu concentration and ratio to Lys to be the as that of control (i.e., 1.63) (optimal + Leu). We hypothesized that 1) lowering CP to meet the minimum SID Leu requirement and Leu:Lys of 1.14, increases dietary energetic efficiency for lactation and reduces heat production associated with lactation compared to a high CP diet with Leu:Lys of 1.63, and 2) supplementation of Leu to the reduced CP diet to meet Leu:Lys of 1.63 reduces dietary energy partitioning towards milk compared to the reduced CP diet with Leu:Lys of 1.14.

## Methods

### Animals and feeding

Fifty-four purebred multiparous (parity 3.4 ± 0.6) Yorkshire sows were selected at day 105 of gestation, balanced by parity and randomly assigned to 1 of 3 dietary treatments [control (CON), *n* = 18; Optimal (OPT), *n* = 19; Optimal + Leu (OPTLEU), *n* = 17]. Sows were moved to conventional farrowing crates and accustomed to their experimental diets beginning at day 105 of gestation. Within the first 24 h of farrowing, litters were equalized to 11 piglets with the objective of weaning 10 piglets per sow. Sows were gradually fed 1.88 kg/d on day 1 to reach 7.44 kg/d on day 21 of lactation according to the NRC model [13], corresponding to an average daily feed intake of 6 kg/d. Sows were provided 3 meals (07:00, 13:00, and 17:00) daily with actual feed intake and feed refusal recorded before each morning meal. Fresh water was available freely for all sows and piglets. Iron injection and surgical castration were conducted on day 1 and 7 post-farrowing, respectively, according to farm protocol. Piglets were not supplied with creep feed. The BW and backfat thickness of sows were recorded on day 1 and 21, and litter weights were recorded on day 1, 4, 8, 14, 18, and 21 according to Zhang et al. [8]. Milk yield was estimated for early (between day 4 and 8) and peak lactation (between day 14 and 18) as previously reported [8].

### Dietary treatments

Ingredients and calculated nutrient composition of the diets are presented in Table 1. Analyzed total and free AA of the diets are presented in Table 2. The NRC model [13] was used to estimate requirements for AA, net energy (NE), calcium (Ca) and phosphorus (P). The requirements were predicted based on the swine herd performance at the Michigan State University Swine Teaching and Research Center, as follows: sow BW of 210 kg, parity number of 2 and above, and daily intake of 6 kg/d, litter size of 10, piglet BW gain of 280 g/d over a 21-day lactation period, and an ambient temperature of 20 °C. The model predicted a minimum sow BW loss of 7.5 kg and the protein:lipid in the model was adjusted to the minimum allowable value of near zero. All diets were formulated to contain the same SID Lys (9 g/kg) and NE (10.80 MJ/kg) concentrations.

**Table 1:** Ingredient composition and nutrient content of control (CON), ptimal (OPT) and Optimal + leucine (OPTLEU) diets (g/kg, as-fed basis)

	CON	OPT	OPTLEU
Ingredient composition			
Corn, yellow dent	591.7	614.5	612.1
Soybean meal^1^	300.0	140.0	140.0
Soy hulls	–	105.7	105.7
Sugar food product^2^	50.0	50.0	50.0
Beef tallow	33.5	50.2	48.1
*L*-Lysine·HCl, 78.8%	–	4.7	4.7
*L*-Valine	–	2.9	2.9
*L*-Threonine	–	2.0	2.0
*L*-Phenylalanine	–	1.3	1.3
*DL*-Methionine	–	1.1	1.1
*L*-Isoleucine	–	0.8	0.8
*L*-Histidine	–	0.7	0.7
*L*-Tryptophan	–	0.5	0.5
*L*-Leucine	–	–	4.5
Limestone	11.8	9.3	9.3
Dicalcium phosphate	4.5	7.8	7.8
Sodium chloride	5.0	5.0	5.0
Vitamin and mineral Premix^3^	2.5	2.5	2.5
Titanium dioxide	1.0	1.0	1.0
Calculated nutrient^4^			
Net energy, MJ/kg	10.8	10.8	10.8
Crude protein	192.4	140.0	143.4
Fermentable fiber	115.8	115.8	115.7
SID^5^ amino acids			
Arginine	11.7	7.1	7.1
Histidine	4.7	3.7	3.7
Isoleucine	7.1	5.2	5.2
Leucine	14.7	10.3	14.7
Lysine	9.0	9.0	9.0
Methionine^6^	2.7	3.0	3.0
Methionine + cysteine	5.4	4.9	4.9
Phenylalanine	8.4	6.7	6.7
Phenylalanine + tyrosine	13.8	10.3	10.3
Threonine	6.1	5.8	5.8
Tryptophan	2.1	1.7	1.7
Valine	7.7	7.9	7.9
Nitrogen	26.3	18.8	19.3
Total calcium^7^	6.5	6.5	6.5
STTD^8^ phosphorus^7^	2.3	2.3	2.3

^1^480 g/kg crude protein

^2^Supplied per kg: net energy 11.9 MJ; fermentable fiber 0.5 g/kg; crude protein 10 g/kg (International Ingredient Corporation, St. Louis, MO, USA)

^3^Sow micro 5 and Se-yeast PIDX15 (Provimi North America, Inc. Brookville, OH, USA)

^4^Based on nutrient concentrations in feed ingredients according to NRC [13]

^5^SID: standardized ileal digestible

^6^Methionine concentration in Optimal and Optimal + Leucine is higher than Control because methionine was added to meet requirement of (methionine + cysteine)

^7^Additional calcium (1.0 g/kg) and phosphorus (1.5 g/kg) releases were accounted due to phytase from the premix

^8^STTD: standard total tract digestible

**Table 2:** Analyzed and calculated concentration of nitrogen, total and free amino acids in control (CON), optimal (OPT) and optimal + leucine (OPTLEU) diets^1^ (g/kg, as-fed basis)

	CON		OPT		OPTLEU	
	Analyzed	Calculated^2^	Analyzed	Calculated^2^	Analyzed	Calculated^2^
Total						
Dry matter	887.6	–	889.5	–	891.5	–
Nitrogen	30.0	30.8	22.0	22.4	22.8	22.9
Arginine	12.3	12.6	7.5	7.8	8.0	7.8
Histidine	4.9	5.3	3.9	4.3	4.0	4.3
Isoleucine	8.5	8.1	6.1	6.0	6.4	6.0
Leucine	16.5	16.7	11.4	11.9	15.9	16.4
Lysine	11.1	10.4	10.8	10.1	11.1	10.1
Methionine	2.7	3.1	2.7	3.3	3.1	3.3
Methionine + cysteine	5.6	6.3	4.8	5.7	5.2	5.7
Phenylalanine	9.8	9.6	7.5	7.6	7.7	7.6
Phenylalanine + tyrosine	16.0	15.9	11.9	12.0	12.3	12.0
Threonine	7.2	7.3	6.4	6.8	6.6	6.8
Tryptophan	2.5	2.3	1.8	1.9	1.8	1.9
Valine	9.4	9.0	8.9	8.9	9.2	8.9
Free amino acids						
Arginine	0.3	–	0.1	–	0.1	–
Histidine	–	–	0.7	0.7	0.7	0.7
Isoleucine	0.1	–	0.8	0.8	0.8	0.8
Leucine	0.1	–	0.1	–	4.3	4.5
Lysine	0.2	–	3.6	3.7	3.7	3.7
Methionine^3^	–	–	0.7	1.1	0.7	1.1
Methionine + cysteine	–	–	0.7	1.1	0.7	1.1
Phenylalanine	–	–	1.2	1.3	1.2	1.3
Phenylalanine + tyrosine	0.1	–	1.2	1.3	1.2	1.3
Threonine	0.2	–	2.0	2.0	2.0	2.0
Tryptophan^4^	–	–	–	0.5	–	0.5
Valine	–	–	2.7	2.9	2.7	2.9

^1^Analyzed values represents average across 3 blocks (feed mixes)

^2^Calculated values for the total amino acids are based on the amino acids concentration in feed ingredients according to NRC [13], and calculated values for the free amino acids correspond to the dietary inclusion rate in crystalline form

^3^Addition of *DL*-Methionine was omitted in one of the 3 blocks, thus reducing the overall free methionine concentration across all 3 blocks. The average free methionine concentration between blocks 1 and 3 was 0.11 and was zero in block 2. Therefore, across blocks 1, 2 and 3, average free Met was 0.07

^4^Analysis of free tryptophan was not performed

The control diet (CON) was formulated using corn and soybean meal as the only sources of Lys to meet NRC [13] SID Lys requirement (9 g/kg) and contained 187.5 g/kg CP. Valine (Val) nearly met SID requirement of 7.7 g/kg (vs. 7.9 g/kg) according to NRC [13]. All other IDAA SID concentrations were in excess relative to NRC [13].

A second diet balanced to reach a NIAA profile was formulated. The NIAA diet was designed by reducing soybean meal relative to corn to meet the minimum SID Leu requirement, which corresponded to a CP concentration of 137.5 g/kg. Then, supplemental crystalline source of *L*-histidine (His), *L*-isoleucine (Ile), *L*-Lys, *DL*-Met, *L*-phenylalanine (Phe), *L*-threonine (Thr), *L*-tryptophan (Trp) and *L*-Val and were added to meet the minimum SID requirement for those AA in the NIAA diet. Crystalline *DL*-methionine was added to meet the requirement of Met + cysteine (Cys). This diet is referred to as the optimal diet (OPT) throughout the remainder of the manuscript.

A third diet was formulated to be the same as OPT with added crystalline *L*-leucine to equate the SID Leu concentration of CON and referred to as OPTLEU. Sugar food product (International Ingredient Corporation, St. Louis, MO, USA) was included in all 3 diets at 50 g/kg to increase diet palatability. Titanium dioxide was included at 1 g/kg as an indigestible index in all diets.

### Energy balance procedure and milk sampling

Energy balance was performed during early lactation (between day 4 and 8) and peak lactation (between day 14 and 18) on a total of 33 sows. Urinary catheter insertion, urine collection and sow milk sampling were carried out as previously reported [8].

### Energy, nutrient and titanium analysis

Feed, fecal and urinary samples were analyzed for gross energy (GE) by bomb calorimetry according to the manufacturer’s instructions (Parr Instrument Inc., Moline, IL, USA). Dry matter, N and titanium in feed and fecal samples were analyzed according to Zhang et al. [8]. Dietary AA analysis [14] was performed by the Agricultural Experiment Station Chemical Laboratories (University of Missouri-Columbia, Columbia, MO, USA) as outlined by Zhang et al. [8]. Whole milk samples were analyzed for fat, true protein, lactose, and milk urea N (MUN) with infrared spectroscopy by the Michigan Dairy Herd Improvement Association (NorthStar Cooperative®, Lansing, MI, USA) [8].

### Calculations

Calculation of body protein (BP) and lipid (BL) composition were predicted by empty body weight (EBW) and backfat [13] using the following equations:

$$ EBW\ (kg)=0.96\times maternal\  BW\ (kg) $$

$$ Maternal\  BL\ (kg)=-26.4\ (kg)+0.221\times maternal\  EBW\ (kg)+1.331\left( kg/ mm\right)\times P2\  backfat\ (mm) $$

$$ Maternal\  BP\ (kg)=2.28\ (kg)+0.178\times maternal\  EBW\ (kg)-0.333\ \left( kg/ mm\right)\times P2\  backfat\ (mm) $$

$$ Maternal\  BL\  or\  BP\  change\ (kg)=d\ 21\  of\ maternal\  BL\  or\  BP\ (kg)-d\ 1\  of\ maternal\  BL\  or\  BP\ (kg) $$

$$ Maternal\  BP\  or\  BL\  Composition\ \left(\%\right)=\frac{Maternal\  BP\  or\  BL\ (kg)}{EBW\ (kg)}\times 100\% $$

Calculation of total and maternal energy retention were performed as follows:

$$ Total\ energy\ retention\ \left( MJ/d\right)= energy\ intake\left( MJ/d\right)- fecal\ energy\ output\left( MJ/d\right)- urinary\ energy\ output\left( MJ/d\right)- energy\ for\ maintenance\left( MJ/d\right) $$

$$ Maternal\ energy\ retention\left( MJ/d\right)= energy\ intake\left( MJ/d\right)- fecal\ energy\ output\left( MJ/d\right)- urinary\ energy\ output\left( MJ/d\right)- energy\ for\ maintenance\left( MJ/d\right)- milk\ energy\ output\left( MJ/d\right) $$

Metabolizable energy (ME) value of diets for maintenance (MJ/kg feed) and ME requirement per day (MJ/d) was calculated based on metabolic body weight (BW^0.75^) as follows:

$$ ME\  for\ maintenance\ \left( MJ/ kg\  feed\right)=\frac{Daily\  ME\  for\ mainteance\ \left( MJ/d\right)}{ Daily\ intake\ \left( kg/d\right)} $$

$$ Daily\  ME\  for\ maintenance\ \left( MJ/d\right)=0.419\times {BW}^{0.75} $$

The NE value of diets for lactation was calculated as follows:

$$ Dietary\  NE\  for\ lactation\ \left( MJ/ kg\  feed\right)= NE\  in\ milk\ \left( MJ/ kg\  feed\right)- NE\  mobilized\ \left( MJ/ kg\  feed\right) $$

Where,

$$ NE\  in\ milk\ \left( MJ/ kg\  feed\right)=\frac{Daily\ energy\ output\ in\ milk\ \left( MJ/d\right)}{Daily\ intake\ \left( kg/d\right)} $$

$$ NE\  mobilized\ \left( MJ/ kg\  feed\right)=\frac{Daily\ energy\ mobilized\ \left( MJ/d\right)}{Daily\ intake\ \left( kg/d\right)} $$

Apparent energy efficiency for milk was calculated as follows:

$$ Apparent\ energy\ efficiency\left(\%\right)=\frac{Milk\ energy\ output\ \left( MJ/d\right)}{Energy\ intake\ or\ absorbed\ \left( MJ/d\right)}\times 100\% $$

Apparent energy efficiency does not account for the milk energy originating from mobilized body pool and energy lost in urine. To determine true energy efficiency for milk, energy mobilized from the body was removed from the daily energy in milk, and energy for maintenance was removed from ME intake as follows:

$$ True\ energy\ efficiency\left(\%\right)=\frac{Daily\ dietary\ energy\ in\ milk\ \left( MJ/d\right)}{Daily\ dietary\  ME\  for\ milk\ \left( MJ/d\right)}\times 100\% $$

Where,

$$ Daily\ dietary\ energy\ in\ milk\left( MJ/d\right)= Daily\ energy\ in\ milk\left( MJ/d\right)- daily\ milk\ energy\ mobilized\ from\ body\ \left( MJ/d\right) $$

$$ Daily\ dietary\  ME\  for\ milk\left( MJ/d\right)= Daily\  ME\  intake\left( MJ/d\right)- daily\  ME\  for\ maintenance\left( MJ/d\right) $$

Energy in milk was calculated by summing energy in milk protein (23.86 kJ/g), fat (39.77 kJ/g) and lactose (16.54 kJ/g), respectively [15]. Energy mobilized from the maternal body was calculated based on change in BP (△BP) and change in BL (△BL) multiplied by 23.44 kJ/g protein and 39.36 kJ/g fat [16], respectively, with an efficiency of body energy mobilization to milk of 0.87 [13], as follows:

$$ Mobilized\ energy\ \left( MJ/d\right)=-\left(\Delta  BP\times 5.7\  MJ/g+\Delta  BL\times 9.4\  MJ/g\right)\times 0.87 $$

A value of 0 was used for mobilized energy when sow body protein and fat depositions were null or positive.

The ME for maintenance was calculated based on NRC [13] as follows:

$$ {ME}_{maintenance}\ \left( MJ/d\right)=0.419\times {BW}^{0.75} $$

The NE for maintenance was calculated based on fasting heat production of sows fed corn-soybean meal basal diet by Wang et al. [17] as follows:

$$ {NE}_{maintenance}\left( MJ/d\right)=0.310\times {BW}^{0.75} $$

The corrected dietary NE (NE_c_) was calculated as follows [18]:

$$ {NE}_c\ \left( MJ/ kg\  feed\ \right)=\frac{NE_{maintenance}\left( MJ/d\right)+ daily\ milk\ energy\left( MJ/d\right)- daily\ mobilized\ energy\ \left( MJ/d\right)}{Daily\ feed\ intake\ \left( kg/d\right)} $$

Heat production associated with lactation was calculated as follows:

$$ Heat\kern0.3em {Production}_{lactation}\kern0.2em \left[ MJ/\left(d\bullet {BW}^{0.75}\right)\right]=\frac{Daily\kern0.2em {heat\kern0.34em production}_{lactation}\left( MJ/d\right)}{Sow\kern0.2em metabolic\kern0.34em body\kern0.40em weight\left({BW}^{0.75}\right)} $$

Where,

$$ Daily\ {Heat\ Production}_{lactation}\ \left( MJ/d\right)= Daily\ dietary\  ME\  for\ milk\left( MJ/d\right)- Daily\ dietary\ energy\ in\ milk\left( MJ/d\right) $$

### Statistical analysis

Statistical analyses were conducted using the mixed model procedure of SAS (SAS Inst. Inc., Cary, NC, USA) according to the following model:

$$ {\mathrm{Y}}_{ij klm}=\upmu +{\mathrm{a}}_i+{\mathrm{b}}_j+{\mathrm{p}}_k+{\mathrm{t}}_l+{\mathrm{d}}_{m(ij)}+{\left(\mathrm{ab}\right)}_{ij}+{\left(\mathrm{ap}\right)}_{ik}+{\left(\mathrm{at}\right)}_{il}+{\mathrm{e}}_{ij klm} $$

where Y_*ijklm*_ is the response on animal *m* of parity k for treatment *i* in block *j* at period *l*, μ is the treatment mean, a_*i*_ is the fixed effect of dietary treatment *i,* p_*k*_ is the fixed effect of parity *k* (e.g. early [P 2–3] vs. late parity [P 4–6]), t_*l*_ is the fixed effect of period *l* (e.g., early vs. peak lactation), b_*j*_ is the fixed effect of block *j,* d_*m(ij)*_ is the random effect of animal *m* nested within treatment *i* and block *j*, (ap)_*ik*_ is the fixed interactive effect of treatment *i* with parity *k,* (at)_*il*_ is the fixed interactive effect of treatment *i* with period *l*, (ab)_*ij*_ is the random interactive effect of treatment *i* with block *j*, and e_*ijklm*_ is the random error on animal *m* of parity *k* for treatment *i* in block *j* at period *l*. When appropriate, a reduced model was used. Specifically, parity and parity × treatment effects were not significant and therefore were not included in the reduced model for analyses of body tissue mobilization, energy balance, energy partitioning, estimated water output, energy efficiency and estimated total heat production. Pairwise comparisons were performed between diets (OPT vs. CON, OPTLEU vs. CON, and OPTLEU vs. OPT) for different periods of lactation (early, peak, and 21-day overall lactation) and between early and peak lactation for each diet using the slice option in SAS and Tukey adjustment. Simple t-test was conducted to compare the analyzed and calculated NE values. Effects were declared significant at *P* ≤ 0.05, and tendencies were declared at 0.05 ≤ *P* ≤ 0.10.

## Results

### Experimental diets

Diet composition and nutrient concentrations are presented in Table 1 and IDAA concentrations are presented in Table 2, as described in Zhang et al. [8].

### Body protein and lipid mobilization

The BP and BL mobilization over 21-day of lactation for all sows are presented in Table 3. Sow BW change, BP and BL mobilization did not differ between treatments. The BW loss and BL mobilization differed from 0 (*P* <  0.05) in sows fed OPT.

**Table 3:** Sow and litter growth performance of sows fed Control (CON; 187.4 g/kg crude protein), Optimal (OPT; 137.8 g/kg crude protein) or Optimal + Leucine (OPTLEU; 142.5 g/kg) diets over a 21-day lactation period^1^

Item	Diet			SEM^2^	*P*-value		
	CON	OPT	OPTLEU		OPT vs. CON	OPT LEU vs. CON	OPTLEU vs. OPT
Number of sows	18	19	17				
Body protein day 1^3^, kg	38.7	38.5	39.0	1.5	0.997	0.962	0.937
Body protein day 21^3^, kg	38.7	38.3	39.4	1.4	0.952	0.876	0.719
Protein mobilization^4^, g/d	5.5	−12.8	21.8	21.0	0.803	0.847	0.497
Protein tissue mobilization^5^, g/d	27.5	−64.0	109.0	105.0	0.803	0.847	0.497
Body lipid day 1^3^, kg	48.1	51.2	51.6	2.0	0.548	0.477	0.985
Body lipid day 21^3^, kg	46.2	44.8	49.4	2.0	0.856	0.465	0.246
Lipid mobilization^4^, g/d	−88.5	− 314.1^*^	− 113.9	74.6	0.143	0.968	0.207
Lipid tissue mobilization^5^, g/d	−106.2	− 376.9^*^	− 136.7	89.5	0.143	0.968	0.207
Sow BW day 1, kg	246	249	252	7	0.921	0.787	0.953
Sow BW day 21, kg	244	241	251	7	0.931	0.724	0.518
Calculated BW change^6^, kg	−1.6	−9.3	−0.6				
Actual BW change, kg	−1.6	−8.3^*^	−0.6	3.0	0.282	0.969	0.216

^1^Data are least squares means

^2^Maximum value of the standard error of the means

^3^Body protein and lipid on day 1 and 21 were predicted based on sow body weight (BW) and backfat loss [13]

^4^Protein and lipid mobilization represent body protein and lipid loss without associated water, and the values were predicted based on sow body weight (BW) and backfat loss [13]

^5^Protein and lipid tissue mobilization represent body protein and lipid loss including the associated water as follows: 1 g of protein is associated with 4 g of water in 5 g of tissue and 1 g of fat is associated with 0.2 g of water in 1.2 g of tissue [16]

^6^Calculated BW change (g) = (protein tissue mobilization + lipid tissue mobilization) × lactation length (21 day)

^*^BW change (*P* = 0.02) and lipid (tissue) mobilization (*P* < 0.01) differed from 0

### Energy balance

Energy balance results are presented in Table 4. In early lactation, urinary and milk energy concentration and output, and total and maternal energy retention did not differ across diets. In peak lactation, urinary energy concentration did not differ across diets. Sows fed OPT had lower urinary energy output (*P* <  0.05) than CON, while sows fed OPTLEU did not differ from either CON or OPT. Sows fed OPT had higher milk energy concentration (*P* <  0.05) and milk energy output (*P* <  0.05) than CON. Sows fed OPTLEU tended to (*P* = 0.07) have less milk energy output than OPT, and did not differ from CON in either milk energy concentration or output. Total energy retention did not differ across diets. Maternal energy retention was lower (*P* <  0.05) in sows fed low protein diets (OPT and OPTLEU) than those fed CON, and did not differ between OPTLEU and OPT.

**Table 4:** Energy balance of sows fed Control (CON; 187.4 g/kg crude protein), Optimal (OPT; 137.8 g/kg crude protein) or Optimal + Leucine (OPTLEU; 142.5 g/kg) diets between day 4 and 8 of lactation (early lactation) and between day 14 and 18 of lactation (peak lactation)^1^

Item	Diet			SEM^2^	*P*-value		
	CON	OPT	OPTLEU		OPT vs. CON	OPT LEU vs. CON	OPTLEU vs. OPT
Early lactation (day 4 to 8)							
Number of sows	12	11	11				
Input							
Feed intake, kg/d	4.9	4.9	4.6	0.2	0.981	0.415	0.530
Energy intake, MJ/d	84.68	83.27	79.82	3.53	0.937	0.475	0.690
Energy absorbed, MJ/d	74.52	72.40	67.81	3.36	0.869	0.268	0.536
Output, kg/d							
Feces (dry matter basis)	0.52	0.54	0.59	0.06	0.980	0.716	0.827
Urine (as-is)	10.68	4.66	5.49	1.68	0.041	0.087	0.930
Milk (as-is)	8.82	8.86	9.51	0.85	0.999	0.762	0.789
Energy concentration, MJ/kg							
Feces (dry matter basis)	19.41	20.20	20.10	0.13	< 0.001	< 0.001	0.756
Urine (as-is)	0.22	0.26	0.26	0.05	0.766	0.791	0.999
Milk (as-is)	4.72	5.10	4.75	0.21	0.170	0.989	0.232
Energy output, MJ/d							
Feces	10.08	10.88	11.79	1.03	0.847	0.477	0.808
Urine	1.68	1.10	1.23	0.28	0.311	0.481	0.948
Milk	41.35	45.38	44.74	3.71	0.718	0.791	0.992
Energy for maintenance, MJ/d^3^	25.94	26.76	26.02	0.59	0.442	0.992	0.502
Total energy retention, MJ/d^4^	46.92	44.66	40.37	3.57	0.926	0.113	0.222
Maternal energy retention, MJ/d^5^	5.84	−0.97	−1.83	3.93	0.434	0.352	0.987
Peak lactation (day 14 to 18)							
Number of sows	11	11	11				
Input							
Feed intake, kg/d	6.8^*^	6.7^*^	6.3^*^	0.2	0.975	0.169	0.242
Energy intake, MJ/d	116.79^*^	114.95^*^	109.64^*^	3.50	0.898	0.216	0.418
Energy absorbed, MJ/d	102.84^*^	100.22^*^	93.08^*^	3.34	0.811	0.073	0.230
Output, kg/d							
Feces (dry matter basis)	0.72^*^	0.74^*^	0.81^*^	0.06	0.969	0.463	0.605
Urine (as-is)	12.04^*^	5.64	6.14	1.68	0.029	0.047	0.974
Milk (as-is)	11.68^*^	13.93^*^	12.13^*^	0.85	0.077	0.893	0.178
Energy concentration, MJ/kg							
Feces (dry matter basis)	19.41	20.20	20.10	0.13	< 0.001	< 0.001	0.756
Urine (as-is)	0.26	0.25	0.31	0.05	0.973	0.750	0.613
Milk (as-is)	4.45	5.03	4.81	0.21	0.027	0.213	0.562
Energy output, MJ/d							
Feces	13.85^*^	14.87^*^	16.33^*^	1.03	0.766	0.223	0.581
Urine	2.50^*^	1.29	1.77^†^	0.28	0.012	0.163	0.446
Milk	51.76^*^	70.21^*^	58.09^*^	3.73	0.005	0.461	0.072
Energy for maintenance, MJ/d^3^	26.26	26.26	26.31	0.59	0.999	0.997	0.996
Total energy retention, MJ/d^4^	74.15^*^	72.51^*^	65.06^*^	3.54	0.926	0.113	0.222
Maternal energy retention, MJ/d^5^	22.51^*^	2.26	7.05^†^	3.92	0.003	0.026	0.668

^1^Data are least squares means

^2^Maximum value of the standard error of the means

^3^Energy required for maintenance (MJ/d) was calculated as 0.42 MJ/kg^0.75^ [13]

^4^Total energy retention = energy intake-fecal energy-urinary energy-maintenance energy

^5^Maternal energy retention = energy intake-fecal energy-urinary energy-maintenance energy-milk energy

^*^Main effect of period (early and late) was significant (*P* < 0.05)

^†^Main effect of period (early and late) tended to be significant: urinary energy output (OPTLEU *P* = 0.054); maternal energy retention (OPTLEU *P* = 0.088)

### Apparent efficiency of nitrogen and energy

Apparent efficiency of N and energy utilization results are presented in Table 5. In early lactation, milk N output relative to ME or NE intake, and apparent energy efficiency for milk did not differ across diets. In peak lactation, milk N output relative to NE intake did not differ across diets. Milk N output relative to ME intake in OPT tended to be higher (*P* = 0.088) than CON, and those in OPTLEU did not differ from either CON or OPT. Sows fed OPT had higher (*P* <  0.05) apparent energy efficiency for milk compared to CON, and sows fed OPTLEU did not differ from either CON or OPT.

**Table 5:** Apparent utilization efficiency of nitrogen and energy of sows fed Control (CON; 187.4 g/kg crude protein), Optimal (OPT; 137.8 g/kg crude protein) or Optimal + Leucine (OPTLEU; 142.5 g/kg) diets between day 4 and 8 of lactation (early lactation) and between day 14 and 18 of lactation (peak lactation)^1^

Item	Diet			SEM^2^	*P*-value		
	CON	OPT	OPTLEU		OPT vs. CON	OPT LEU vs. CON	OPTLEU vs. OPT
Early lactation (day 4 to 8)							
Number of sows	12	11	11				
Nitrogen (N) utilization efficiency^3^							
Milk N output/ME intake, g/MJ^4^	0.88	0.90	0.94	0.06	0.960	0.759	0.907
Milk N output/NE intake, g/MJ^4^	1.17	1.18	1.24	0.08	0.997	0.824	0.869
Energy utilization efficiency							
Total energy retention, % of energy intake	55.1	53.3	50.8	1.6	0.703	0.163	0.537
Total energy retention, % of energy absorbed	62.6	61.5	59.6	1.6	0.847	0.298	0.606
Milk energy output, % of energy intake	49.5	55.2	54.6	3.7	0.529	0.599	0.993
Milk energy output, % of energy absorbed	56.2	63.4	63.6	4.4	0.461	0.442	0.999
Peak lactation (day 14 to 18)							
Number of sows	11	11	11				
Nitrogen (N) utilization efficiency^3^							
Milk N output/ME intake, g/MJ^4^	0.86	1.05^*^	0.93	0.06	0.088	0.660	0.384
Milk N output/NE intake, g/MJ^4^	1.14	1.38^*^	1.23	0.09	0.115	0.730	0.394
Energy utilization efficiency							
Total energy retention, % of intake	63.2^*^	62.8^*^	58.6^*^	1.6	0.986	0.140	0.187
Total energy retention, % of absorbed	71.8^*^	72.2^*^	69.1^*^	1.6	0.973	0.369	0.265
Milk energy output, % of energy intake	44.5	62.3	53.0	3.7	0.007	0.268	0.199
Milk energy output, % of energy absorbed	50.7	71.5	62.2	4.4	0.006	0.167	0.304

^1^Data are least squares means

^2^Maximum value of the standard error of the means

^3^Milk N = Milk true protein × 6.25 + milk urea N

^4^The ME and NE intake were based on calculated values of ME and NE

^*^Main effect of period (early and late) was significant (*P* < 0.05)

### Dietary energy partitioning

Dietary energy partitioning is presented in Tables 6 and 7. In both early and peak lactation (Table 6), digestible energy (DE) value of low protein diets was lower (*P* <  0.01; OPTLEU) or tended to be lower (*P* = 0.06; OPT) than that of CON. The DE value of OPTLEU did not differ from OPT. The ME values of all diets did not differ. The NE_c_ value did not differ between diets in early lactation and tended to be higher (*P* = 0.09) in OPT compared to CON in peak lactation. Compared to the calculated NE values, the NE_c_ value was higher (*P* < 0.05) in OPTLEU during early lactation and tended to be higher (*P* = 0.06) in OPT during peak lactation.

**Table 6:** Dietary energy partitioning of sows fed Control (CON; 187.4 g/kg crude protein), Optimal (OPT; 137.8 g/kg crude protein) or Optimal + Leucine (OPTLEU; 142.5 g/kg) diets between day 4 and 8 of lactation (early lactation) and between day 14 and 18 of lactation (peak lactation)^1^

Item	Diet			SEM^2^	*P*-value		
	CON	OPT	OPTLEU		OPT vs. CON	OPT LEU vs. CON	OPTLEU vs. OPT
Early lactation (day 4 to 8)							
Number of sows	12	11	11				
Feed intake, kg/d	4.9	4.9	4.6	0.10	0.899	0.102	0.135
Gross energy (GE), MJ/kg							
Analyzed	17.24	17.10	17.33	–	–	–	–
Calculated	17.22	17.58	17.57				
Digestible energy (DE), MJ/kg							
Analyzed	15.22	14.90	14.77	0.10	0.062	0.006	0.571
Calculated	15.03	14.70	14.71				
Metabolizable energy (ME), MJ/kg							
Analyzed	14.84	14.64	14.52	0.20	0.766	0.507	0.904
Calculated	14.44	14.26	14.26				
Corrected net energy (NE_c_), MJ/kg^3^	11.56	11.36	12.63	0.69	0.977	0.511	0.405
NE_lactation_^4^	7.65	7.28	8.57	0.71	0.928	0.625	0.417
NE_maintenance_^5^	3.91	4.07	4.16	0.15	0.536	0.233	0.822
Calculated	10.80	10.80	10.80				
Peak lactation (day 14 to 18)							
No. of sows	11	11	11				
Feed intake, kg/d	6.8	6.7	6.3	0.1	0.865	0.034	0.049
Gross energy (GE), MJ/kg							
Analyzed	17.24	17.10	17.33	–	–	–	–
Calculated	17.22	17.58	17.57				
Digestible energy (DE), MJ/kg							
Analyzed	15.22	14.90	14.77	0.10	0.062	0.006	0.571
Calculated	15.03	14.70	14.71				
Metabolizable energy (ME), MJ/kg							
Analyzed	14.81	14.67	14.45	0.20	0.887	0.427	0.709
Calculated	14.44	14.26	14.26				
Corrected net energy (NE_c_), MJ/kg^3^	10.01^*^	12.16	11.25	0.69	0.087	0.418	0.623
NE_lactation_^4^	7.13	9.26^*^	8.13	0.71	0.105	0.584	0.506
NE_maintenance_^5^	2.88^*^	2.93	3.14	0.15	0.952	0.221	0.349
Calculated	10.80	10.80	10.80				

^1^Data are least squares means; energy is presented as MJ/kg feed

^2^Maximum value of the standard error of the means

^3^ NE_c_(MJ/kg feed ) = NE_milk_(MJ/kg feed) + NE_maintenance_(MJ/kg feed). NE was higher (*P* < 0.05) than calculated NE in OPTLEU diet during early lactation and tended to be higher (*P* = 0.057) in OPT diet during peak lactation

^4^ ${\mathrm{NE}}_{\mathrm{lactation}}\left(\mathrm{MJ}/\mathrm{kg}\ \mathrm{feed}\ \right)=\frac{\mathrm{Milk}\ \mathrm{energy}\ \mathrm{output}\left(\mathrm{MJ}/\mathrm{d}\right)-\mathrm{Milk}\ \mathrm{energy}\ \mathrm{from}\ \mathrm{body}\ \left(\mathrm{MJ}/\mathrm{d}\right)}{\mathrm{Daily}\ \mathrm{feed}\ \mathrm{intake}\left(\mathrm{kg}/\mathrm{d}\right)}$. NE_lactation_ was lower than (*P* < 0.01) calculated NE in each experimental diet during both early and peak lactation

^5^$$ {\mathrm{NE}}_{\mathrm{maintenane}}\left(\mathrm{MJ}/\mathrm{kgfeed}\right)=\frac{0.310\times {\mathrm{BW}}^{0.75}\left(\mathrm{MJ}/\mathrm{d}\right)}{\mathrm{Daily}\kern0.34em \mathrm{feed}\kern0.34em \mathrm{intake}\left(\mathrm{kg}/\mathrm{d}\right)} $$

^*^Main effect of period (early and late) was significant (*P* < 0.05).

**Table 7:** The relative values between dietary gross energy (GE), digestible energy (DE), metabolizable energy (ME), and net energy (NE) of sows fed Control (CON; 187.4 g/kg crude protein), Optimal (OPT; 137.8 g/kg crude protein) or Optimal + Leucine (OPTLEU; 142.5 g/kg) diets between day 4 and 8 of lactation (early lactation) and between day 14 and 18 of lactation (peak lactation)^1^

Item	Diet			SEM^2^	*P*-value		
	CON	OPT	OPTLEU		OPT vs. CON	OPT LEU vs. CON	OPTLEU vs. OPT
Early lactation (day 4 to 8)							
Number of sows	12	11	11				
DE/GE, %							
Analyzed	88.3	87.2	85.2	0.4	0.162	< 0.01	0.007
Calculated	87.3	83.6	83.7				
ME/DE, %							
Analyzed	97.7	98.5	98.2	0.4	0.324	0.656	0.836
Calculated	96.0	97.0	97.0				
NE_lactation_/ME, %^3^							
Analyzed	51.4	49.7	58.9	4.8	0.967	0.507	0.380
Calculated	74.8	75.8	75.7				
NE_milk_/ME, %^4^	57.5	64.4	65.0	4.3	0.500	0.448	0.996
Peak lactation (day 14 to 18)							
Number of sows	11	11	11				
DE/GE, %							
Analyzed	88.3	87.2	85.2	0.4	0.162	< 0.01	0.007
Calculated	87.3	83.6	83.7				
ME/DE, %							
Analyzed	97.5	98.7	98.0	0.4	0.063	0.635	0.327
Calculated	96.0	97.0	97.0				
NE_lactation_/ME, %^3^							
Analyzed	48.0	63.0^*^	56.2	4.8	0.092	0.468	0.584
Calculated	74.8	75.8	75.7				
NE_milk_/ME, %^4^	51.9	72.4	63.5	4.4	0.008	0.167	0.339

^1^Data are least squares means

^2^Maximum value of the standard error of the means

^3^$$ {\mathrm{NE}}_{\mathrm{lactation}}\left(\mathrm{MJ}/\mathrm{kg}\ \mathrm{feed}\ \right)=\frac{\mathrm{Milk}\ \mathrm{energy}\ \mathrm{output}\left(\mathrm{MJ}/\mathrm{d}\right)-\mathrm{Milk}\ \mathrm{energy}\ \mathrm{from}\ \mathrm{body}\ \left(\mathrm{MJ}/\mathrm{d}\right)}{\mathrm{Daily}\ \mathrm{feed}\ \mathrm{intake}\left(\mathrm{kg}/\mathrm{d}\right)} $$

^4^$$ {\mathrm{NE}}_{\mathrm{milk}}\left(\mathrm{MJ}/\mathrm{kg}\ \mathrm{feed}\ \right)=\frac{\mathrm{Milk}\ \mathrm{energy}\ \mathrm{output}\left(\mathrm{MJ}/\mathrm{d}\right)}{\mathrm{Daily}\ \mathrm{feed}\ \mathrm{intake}\left(\mathrm{kg}/\mathrm{d}\right)} $$

^*^Main effect of period (early and late) was significant (*P* < 0.05).

The energy values of NE, ME, DE expressed relative to ME, DE and GE, respectively, are presented in Table 8. In early lactation, the ME/DE, NE_lactation_/ME, and NE_milk_/ME did not differ across diets. In peak lactation, the ME/DE tended to be higher (*P* = 0.063) in OPT than CON. The ME/DE in OPTLEU did not differ from either CON or OPT. Compared to CON, the NE_milk_/ME and NE_lactation_ /ME was higher (*P* < 0.01) or tended to be higher (*P* = 0.092), respectively, in OPT. The NE_milk_/ME and NE_lactation_/ME in OPTLEU did not differ from either CON or OPT. In both early and peak lactation, the DE/GE did not differ between CON and OPT, and was lower (*P* < 0.01) in sows fed OPTLEU than those fed CON or OPT. The NE_lactation_/ME did not differ across diets.

**Table 8:** True energy efficiency and heat production associated with milk production of sows fed Control (CON; 187.4 g/kg crude protein), Optimal (OPT; 137.8 g/kg crude protein) or Optimal + Leucine (OPTLEU; 142.5 g/kg) diets in early (day 4 to day 8), peak (day 14 to day 18) and overall (day 1 to day 21) lactation period^1^

Item	Diet			SEM^2^	*P*-value		
	CON	OPT	OPTLEU		OPT vs. CON	OPT LEU vs. CON	OPTLEU vs. OPT
Early lactation (day 4 to 8)							
Number of sows^3^	12	11	11				
ME_milk_, MJ/d^4^	46.89	44.65	40.35	3.56	0.864	0.306	0.595
ME_intake_	72.77	71.29	66.52	3.45	0.944	0.375	0.564
ME_maintenance_	25.94	26.76	26.02	0.58	0.442	0.992	0.502
Milk energy output from diet, MJ/d^5^	37.40	35.91	41.06	4.54	0.970	0.835	0.705
Milk energy output	41.35	45.38	44.74	3.71	0.718	0.791	0.992
Milk energy output from body	0.46	8.3	2.01	3.04	0.992	1.000	0.995
True energy efficiency, %^6^	79.9	78.8	94.0	7.0	0.993	0.333	0.293
Milk energy from diet^7^	89.8	77.2	90.6	4.7	0.167	0.992	0.132
Milk energy from body	10.2	22.8	9.4	4.7	0.167	0.992	0.132
Heat production associated with lactation^8^, kJ/(d·BW^0.75^)	160	132	30	64	0.944	0.319	0.504
Peak lactation (day 14 to 18)							
Number of sows^3^	11	11	11				
ME_milk_, MJ/d^4^	74.13^*^	72.51^*^	65.06^*^	3.56	0.928	0.114	0.222
ME_intake_	100.30^*^	98.96^*^	91.31^*^	3.45	0.955	0.144	0.238
ME_maintenance_	26.26	26.26	26.31	0.58	0.999	0.997	0.996
Milk energy output from diet, MJ/d^5^	47.98^*^	61.44^*^	52.34^*^	4.54	0.112	0.780	0.347
Milk energy output	51.76^*^	70.21^*^	58.09^*^	3.73	0.005	0.461	0.072
Milk energy output from body	0.47	8.25^*^	2.00	3.04	0.992	1.000	0.995
True energy efficiency, %^6^	65.2^†^	86.1	79.5^†^	7.0	0.106	0.329	0.786
Milk energy from diet^7^	90.6	86.8^*^	90.1	4.7	0.837	0.997	0.869
Milk energy from body	9.4	13.2^*^	9.9	4.7	0.837	0.997	0.869
Heat production associated with lactation, kJ/(d·BW^0.75^)^8^	414^*^	165	211^*^	64	0.028	0.082	0.870
Over a 21-day lactation							
Number of sows^3^	11	9	9				
ME_milk_, MJ/d^4^	60.79	59.15	52.25	3.68	0.924	0.229	0.349
ME_intake_	86.81	85.89	78.39	3.62	0.978	0.264	0.335
ME_maintenance_	26.07	26.74	25.95	0.64	0.670	0.987	0.617
Milk energy output from diet, MJ/d^5^	43.11	48.43	44.38	5.50	0.736	0.982	0.843
Milk energy output	46.93	59.34	49.56	4.91	0.153	0.873	0.272
Milk energy output from body	−0.62	9.40	1.67	3.82	0.241	0.900	0.410
True energy efficiency, %^6^	70.5	82.2	83.2	6.3	0.439	0.390	0.993
Milk energy from diet^7^	91.6	81.6	88.3	5.3	0.425	0.898	0.668
Milk energy from body	8.5	18.4	11.7	5.3	0.425	0.898	0.668
Heat production associated with lactation, kJ/(d·BW^0.75^)^8^	289	154	134	60	0.321	0.248	0.970

^1^Data are least squares means

^2^Maximum value of the standard error of the means

^3^Sows with an actual feed intake as percentage of predicted > 75% during days 4–8 and days 14–18; for over 21-day lactation, sows have energy balance for both early and peak lactation were used

^4^Metabolizable energy (ME) : ME_milk_(MJ/d) = ME_intake_(MJ/d) − ME_maintenance_(MJ/d)

^5^Milk energy output from diet(MJ/d) = Milk energy output(MJ/d) − Milk energy output from body(MJ/d)

^6^$$ \mathrm{True}\ \mathrm{energy}\ \mathrm{efficiency}\left(\%\right)=\frac{\mathrm{Milk}\ \mathrm{energy}\ \mathrm{output}\ \mathrm{from}\ \mathrm{diet}\left(\mathrm{MJ}/\mathrm{d}\right)}{{\mathrm{ME}}_{\mathrm{milk}}\left(\mathrm{MJ}/\mathrm{d}\right)}\times 100\% $$

^7^$$ \mathrm{Milk}\ \mathrm{energy}\ \mathrm{from}\ \mathrm{diet}\left(\%\right)=\frac{\mathrm{Milk}\ \mathrm{energy}\ \mathrm{output}\ \mathrm{from}\ \mathrm{diet}\left(\mathrm{MJ}/\mathrm{d}\right)}{\mathrm{Milk}\ \mathrm{energy}\left(\mathrm{MJ}/\mathrm{d}\right)} $$

^8^$$ \mathrm{Heat}\kern0.34em \mathrm{production}\kern0.34em \mathrm{associated}\kern0.34em \mathrm{with}\kern0.34em \mathrm{lactation}\left[\mathrm{MJ}/\left(\mathrm{d}\bullet {\mathrm{BW}}^{0.75}\right)\right]=\frac{{\mathrm{ME}}_{\mathrm{milk}}\left(\mathrm{MJ}/\mathrm{d}\right)-\mathrm{milk}\ \mathrm{energy}\ \mathrm{output}\ \mathrm{from}\ \mathrm{diet}\left(\mathrm{MJ}/\mathrm{d}\right)}{{\mathrm{BW}}^{0.75}} $$

^*^Main effect of period (early and late) was significant (*P* < 0.05).

^†^Main effect of period (early and late) tended to be significant for true energy efficiency (CON *P* = 0.086; OPTLEU *P* = 0.100).

### Energy efficiency and estimated heat production associated with lactation

True energy efficiency and estimated heat production associated with lactation are presented in Table 8. In early lactation, heat production did not differ across diets. In peak lactation, compared to CON*,* heat production was lower (*P* < 0.05) or tended to be lower (*P* = 0.082) in sows fed OPT and OPTLEU, respectively, and did not differ between OPT and OPTLEU*.* In both early and peak lactation, true milk energy efficiency did not differ across diets. Over 21-day lactation period, true milk energy efficiency and heat production did not differ across diets.

## Discussion

In a previous study conducted on lactating sows [8], we reported that reducing dietary protein to meet the minimum SID Leu requirement increased utilization efficiency of N, arginine (Arg), His, Ile, Leu, Phe + tyrosine (Tyr) and Trp for milk yield while maintaining overall lactation performance. Supplementing Leu to the reduced CP diet did not impact the efficiency of IDAA utilization but appeared to repartition nutrients away from the mammary gland. In the current work we aimed at determining dietary energetic efficiency, partitioning, and heat production associated with lactation in sows fed a reduced protein diet with a NIAA profile (OPT) and OPT diet with supplemental Leu (OPTLEU). In the present study, the term “near ideal AA profile” was chosen in lieu of the conventional “ideal AA profile” because the “ideal AA profile” is conceptual rather than biologically factual. The rationale is further based on the notions that an “ideal AA profile” 1) cannot be limited to the relative contribution of only two AA pools (i.e., milk and maintenance), 2) needs accurate characterization of the maintenance AA pool for the lactating sow, and 3) should include AA for which dietary essentiality is known for lactating sow [i.e., arginine (Arg) and histidine].

The loss of BW in sows fed OPT was mainly associated with BL rather than BP loss, and BL loss was consistently greater than BP loss across diets, implying that the drive for tissue mobilization was likely due to a deficit in energy supply as suggested by others [19, 20]. Mobilization of BL, which is energy dense compared to protein [16], is more efficient than mobilization of BP to satisfy the energy need for milk production. In fact, milk fat content of sows fed OPT was greater [8], further supporting that the increased BW loss was associated mainly with BL. Sows generally lose more BL than BP throughout lactation [21]. It was reported that [18] the loss of BW in lactating sows fed diets containing CP from 146 to 186 g/kg was due to BL mobilization. On the other hand, Huber et al. [6] reported that sows fed a similar low CP diet as this study lost BW over a 21-day lactation period, and indicated based on loin eye area measurements that the BW loss resulted from greater body protein as opposed to BL mobilization. The greater BP loss in that study may have been associated with feeding diets marginally deficient in Lys [6]. In contrast, sows fed CON and OPTLEU lost a minimal amount of BW and were in a positive maternal N balance [8]. This observation suggested that Leu to Lys of 1.63 may impact partitioning of DE by directing energy away from mammary gland and towards the maternal pool.

In this study, mobilization of BP and BL of sows fed OPT (64 and 377 g/d, respectively) and CON (28 and 106 g/d, respectively) was quantified. Our BP mobilization values are in the range reported by Pedersen et al. [17] (i.e., 28 to 64 g/d vs. 20 to 40 g/d) for sows fed CP diets ranging from 146 to 186 g/kg, but those for BL were noticeably lower (i.e., 106 to 377 g/d vs. 800 to 820 g/d). It is unclear whether estimation of BL and BP mobilization by Pedersen et al. [18] was associated with water or not. In this study, BL and BP were quantified with or without water. The other possible reason may be ascribed to a different prediction approach. The current study predicted BP and BL based on sow BW and P2 backfat thickness equations outlined in NRC [13], while Pedersen et al. [18] included deuterium oxide (D_2_O) space in addition to sow BW and P2 backfat thickness [21]. Earlier on, Pedersen et al. [22] estimated BL and BP relative to BW. Their values were 15.7% and 26.8% for BP and BL, respectively, on day 3 of lactation, and 16.7% and 20.9% for BP and BL, respectively, on day 28 of lactation. In our study, on day 1 of lactation, BP and BL were 15.7% and 19.6%, respectively, for CON, and 15.5% and 20.6%, respectively, for OPT. On day 21 of lactation, BP and BL were 15.9% and 18.9%, respectively, for CON, and 15.9% and 18.6%, respectively, for OPT. Again, the predictions of BP % are fairly close between our study and those of Pedersen et al. [22], but those of BL% are lower. It is possible that the approach of NRC [13] may yield lower BL prediction than that of Rozeboom et al. [21]. Litter gain (22.4% and 23.4% greater) and therefore lactation energy demand was considerably greater in both studies by Pedersen et al. [18, 22], compared to that of the current study reported by Zhang et al. [8]. With the advancement of lactation, BL decreased by 5.9% [22] from day 3 to 28, and in our study, BL% decreased by 0.7% and 2% in CON and OPT, respectively, from day 1 to 21.

Feeding the OPT diet improved apparent and true energy utilization efficiency as well as milk N output efficiency relative to ME intake in peak lactation. Total energy retained was similar across diets, but sows fed OPT retained less maternal energy, suggesting that OPT diet resulted in more energy partitioning for milk production. Huber et al. [6] indicated that reduced protein diets favored partitioning of AA towards milk protein yield rather than maternal protein pool. This observation may be in part related to a reduced dietary Leu intake, because Leu stimulates maternal body protein gain [23, 24]. The decreased milk energy output in OPTLEU compared to OPT during peak lactation combined with no differences in total energy retention across dietary treatments implied that additional Leu above requirement may reduce dietary energy partitioning towards milk. This observation is in line with previously reported performance and N balance where sows fed CON and OPTLEU did not lose as much BW as OPT and were in a positive maternal N balance [8].

**Fig. 1:**
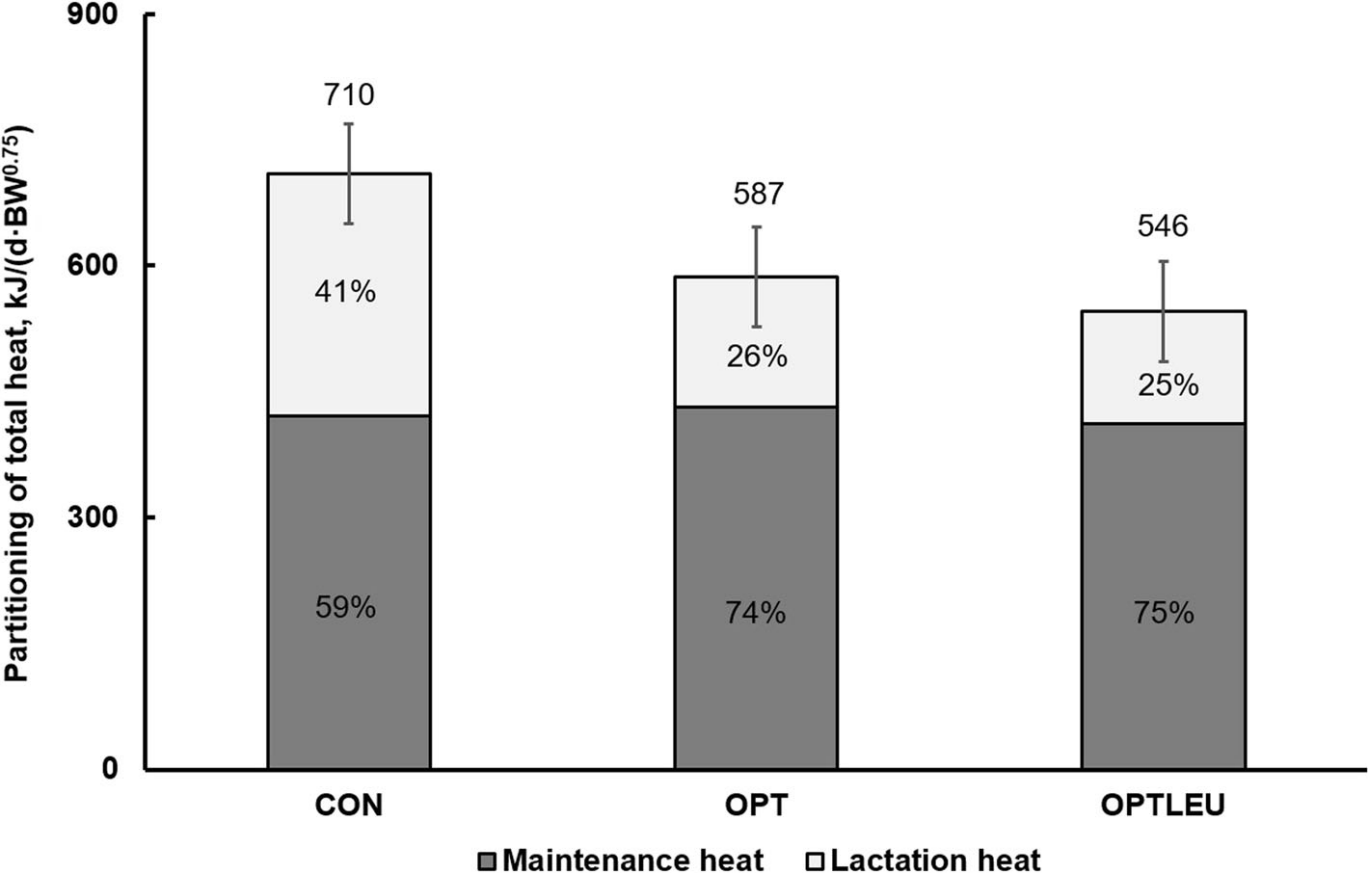
The partitioning of total heat production of sows fed control (CON), optimal (OPT) and optimal + leucine (OPTLEU) over a 21-day lactation period. Total heat production did not differ between diets

The higher NE:ME and ME:DE in peak lactation for OPT fed sows aligns with their improved apparent energy efficiency in peak lactation compared to CON. In addition, the lack of difference in DE:GE in peak lactation indicates that the improvement in apparent efficiency likely occurred during the post-absorptive stage in peak lactation. By definition, urinary energy loss and heat increment represents the difference between “DE to ME” and “ME to NE” [16], suggesting that the improved apparent energetic efficiency in OPT in peak lactation was due to less urinary energy and metabolic heat loss [4, 18]. In fact, urinary energy loss and estimated heat production associated with lactation in the current study was lower in OPT than CON during the peak lactation period. Other studies on growing-finishing pigs [4, 25] and lactating sows [6, 8] showed that urinary N loss decreased by reducing dietary protein. Considering the major contributor of urinary energy is urinary N, primarily from urea [13], less urinary N loss also implies less urinary energy loss. Previous research in growing pigs also showed a 6.7% or 100 kJ/(d·BW^0.65^) decrease in heat production associated with feeding lower dietary CP [4]. During the entire lactation period, the estimated heat associated with lactation was 289, 154, and 134 kJ/(d·BW^0.75^) for CON, OPT and OPTLEU, respectively, corresponding to a 46.7% or 135 kJ/(d·BW^0.75^) reduction in heat between CON and OPT. Note that the total heat production (maintenance + lactation; Fig. 1) added up to be 710, 587, and 546 kJ/(d·BW^0.75^) for CON, OPT and OPTLEU, respectively. Those values fall within range of a previously reported value of 669 kJ/(d·BW^0.75^) measured by indirect calorimetry and respiratory quotient (RQ)-method to separate heat between sow and litter [26]. Recently, Pedersen et al. [18] estimated heat production (maintenance + lactation) based on milk energy output and a constant lactation efficiency of 0.78 and reported values varying between 757 and 803 kJ/(d·BW^0.75^). In our study, the energy efficiency for lactation improved by decreasing dietary CP and with advancement of lactation. Pedersen et al. [18] did not observe a clear trend in total heat production as dietary CP content decreased, similar to this study. However, our results pointed to less lactation heat as percentage of total heat in OPT (26%) and OPTLEU (25%) compared to CON (41%). Our values and those of Pedersen et al. [18] are theoretical estimates and therefore further testing of the impact of dietary CP concentrations in lactating sows on heat production using indirect calorimetry is needed.

Sow milk energy is partially derived from the diet and partially from the maternal body pool. Dietary energy contribution to milk increased from 77% to 87% only in OPT diet as lactation progressed, indicating that the reduced dietary protein with NIAA profile may improve dietary energy partitioning towards milk with advancement of lactation. However, we acknowledge that body mobilization was estimated over a 21-day lactation period, and it was assumed that mobilization rate (g/d) remained constant throughout lactation. Previous studies [20, 27] indicated that lactating sows mobilized greater amounts of body nutrients in early lactation compared to peak lactation. Similarly, in the present study, sows fed OPT had a negative maternal energy retention (− 0.97 MJ/d for OPT and −1.83 MJ/d for OPTLEU) in early lactation only.

The true efficiency value for sows fed CON for a 21-day lactation period was 70.5%, which is fairly close to the estimated NRC [13] value of 72% for sows fed conventional diets meeting the minimum SID Lys requirement. The true efficiency values of 82% and 83% for sows fed OPT and OPTLEU, respectively, did not differ statistically from CON value of 70.5%, presumably due to the variability associated with body weight loss. Nonetheless, future implementation of those values may impact prediction of energy requirement since the energy prediction model of NRC uses a value of 72% [13]. Therefore, additional work is needed with a higher number of animals to verify these values and determine whether NIAA diet increases true energy efficiency. The efficiency value of 78% reported by Pedersen et al. [18] is also higher than NRC [13]. The decrease in true energy efficiency as lactation progressed for CON (79.9% to 65.2%) and OPTLEU (94% to 79.5%) suggests a potential negative effect of Leu on dietary energy partitioning towards milk, whereby Leu directs dietary energy away from the mammary gland and towards the maternal body. A true efficiency value of 94% for sows fed OPTLEU in early lactation is somewhat high and puzzling. Note that no higher apparent efficiency was observed, and maternal mobilization was only taken into account in the calculation of true efficiency. Thus, it was likely due to the assumption of constant maternal mobilization, which may be overestimated in early lactation [20, 27]. Nonetheless, efficiency values reported herein for sows fed CON and OPT are within range of other reported values [13, 18].

Despite that all three experimental diets were formulated iso-calorically based on the NE system (10.80 MJ/kg), the measured NE_c_ (maintenance + lactation) was higher than the calculated values in the low protein diets (OPT or OPTLEU). The present study corrected the NE by excluding the milk energy mobilized from maternal body, because NE is the reflection of dietary energy only [13]. The NE_c_ (maintenance + lactation) was previously estimated [18], but the difference between calculated NE and measured NE_c_ was not statistically compared. A variation of NE_c_ between diets with graded levels of CP was observed and peaked at CP of 156 g/kg [18]. Similarly, the measured NE_c_ in the current study was higher in OPT (138 g/kg CP) than CON (187 g/kg CP) during peak lactation. Note that the measured NE only for lactation (NE_lactation_) in the present study were consistently lower than the calculated values (10.80 MJ/kg) across all diets. Also, NE_lactation_ increased as lactation progressed only in the OPT diet, as reported by Pedersen et al. [18] for NE_c_. Such observation raises question regarding the adequacy of the book value of NE for lactating sows which were derived from growing-finishing pigs according to NRC [13]. In fact, sows utilize dietary energy more efficiently for lactation than growing pigs for retention [18]. Whether the calculated NE [13] corresponded to the sum of maintenance and lactation or lactation alone is unclear and either of them differ from the calculated values. The INRA reported NE values of various feed ingredient for both growing pigs and sows, which might serve as additional reference to diet formulation using NE system [28]. However, NE values generated from INRA are based on predictions from nutrient composition and used for both growing pigs and sows. In fact, difference in NE values for growing pigs and sows are carried over by DE and ME, which are predicted separately for growing pigs and sows. Current results also suggest that NE values for lactating sows are dynamic and dependent on diet (e.g. dietary CP level and AA balance) and stage of lactation of the sow, warranting the need for additional research on the NE system for lactation.

## Conclusion

Feeding a NIAA diet improved the apparent dietary energy utilization due to less urinary energy and metabolic heat loss, a response that was associated with the peak stage of lactation. The estimated value for heat reduction was 154 kJ/(d·BW^0.75^) in sows fed a NIAA diet during peak lactation. Feeding a NIAA diet also triggered dietary energy deposition into milk at the expense of maternal mobilization, implying higher energy requirement when feeding sows with NIAA diet. Leucine supplementation above requirement may reduce dietary energy utilization for lactation by directing dietary energy away from mammary gland and towards maternal pool, partially explaining the effectiveness in energy efficiency of NIAA diet over high CP diets. The estimated heat production values in this study may need to be validated with indirect calorimetry.

## Data Availability

The datasets used and/or analyzed during the current study are available from the corresponding author on request.

## Abbreviations

AA: Amino acid
BW: Body weight
CP: Crude protein
DE: Digestible energy
GE: Gross energy
IDAA: Indispensable amino acid
ME: Metabolizable energy
NE: Net energy
NIAA: Near ideal amino acid profile
SID: Standardized ileal digestibility
